# Yellow Fever: Integrating Current Knowledge with Technological Innovations to Identify Strategies for Controlling a Re-Emerging Virus

**DOI:** 10.3390/v11100960

**Published:** 2019-10-17

**Authors:** Robin D.V. Kleinert, Eduardo Montoya-Diaz, Tanvi Khera, Kathrin Welsch, Birthe Tegtmeyer, Sebastian Hoehl, Sandra Ciesek, Richard J.P. Brown

**Affiliations:** 1 Division of Veterinary Medicine, Paul-Ehrlich-Institute, 63225 Langen, GermanyEduardo.MontoyaDiaz@pei.de (E.M.-D.); 2 Department of Gastroenterology and Hepatology, Faculty of Medicine, University Hospital Essen, University of Duisburg-Essen, 45147 Essen, Germany; Tanvi.Khera@uk-essen.de; 3 University of Veterinary Medicine Hannover, 30559 Hannover, Germany; Kathrin.Welsch@tiho-hannover.de; 4 Institute for Experimental Virology, TWINCORE, Centre for Experimental and Clinical Infection Research; a joint venture between the Medical School Hannover (MHH) and the Helmholtz Centre for Infection Research (HZI), 30625 Hannover, Germany; Birthe.Tegtmeyer@twincore.de; 5 Institute of Medical Virology, University Hospital Frankfurt, 60596 Frankfurt am Main, Germany, Sandra.Ciesek@kgu.de (S.C.)

**Keywords:** yellow fever virus, flavivirus entry factor, global diversity, yellow fever virus tropism, animal models, transmission and vector control, vaccine, host immune response, E protein structure, re-emerging virus

## Abstract

Yellow fever virus (YFV) represents a re-emerging zoonotic pathogen, transmitted by mosquito vectors to humans from primate reservoirs. Sporadic outbreaks of YFV occur in endemic tropical regions, causing a viral hemorrhagic fever (VHF) associated with high mortality rates. Despite a highly effective vaccine, no antiviral treatments currently exist. Therefore, YFV represents a neglected tropical disease and is chronically understudied, with many aspects of YFV biology incompletely defined including host range, host–virus interactions and correlates of host immunity and pathogenicity. In this article, we review the current state of YFV research, focusing on the viral lifecycle, host responses to infection, species tropism and the success and associated limitations of the YFV-17D vaccine. In addition, we highlight the current lack of available treatments and use publicly available sequence and structural data to assess global patterns of YFV sequence diversity and identify potential drug targets. Finally, we discuss how technological advances, including real-time epidemiological monitoring of outbreaks using next-generation sequencing and CRISPR/Cas9 modification of vector species, could be utilized in future battles against this re-emerging pathogen which continues to cause devastating disease.

## 1. General Introduction

Yellow fever was historically the first described viral hemorrhagic fever (VHF) and its causative agent, the yellow fever virus (YFV), is the prototypic member of the family *Flaviviridae*. Pioneering experimental work in the 19th century identified the *Aedes aegypti* mosquitos as the vector responsible for transmission to humans, facilitating subsequent eradication from Europe and North America [1,2,3,4]. However, today, YFV remains prevalent in tropical regions of Africa and South America [4,5], with over 900 million people currently living in endemic areas, 500 million of whom reside in Africa [6]. YFV represents a zoonotic pathogen and complete eradication in tropical regions is not achievable due to rainforest circulation in primate reservoirs [4,6,7]. A highly effective live-attenuated vaccine, the 17D strain, was developed in the 1930s and still in use today, over 80 years later [3,6,8]. Indeed, vaccination represents one of the core strategies employed to stop YFV outbreaks or minimize their impact when they do occur [9].

**Figure 1:**
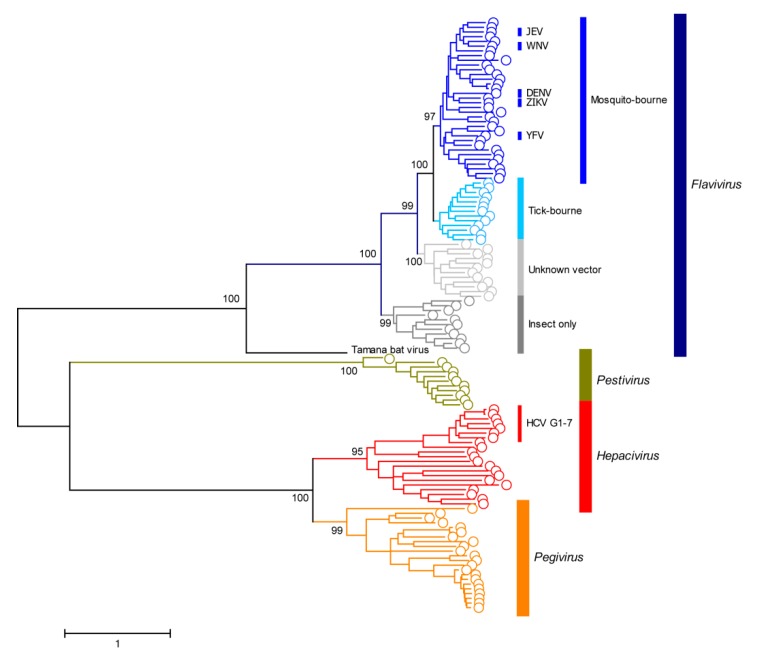
Phylogenetic relationships within the *Flaviviridae*. The evolutionary tree presented was generated using MEGA7 [11] from a conserved region of the viral RNA dependant RNA polymerase (RdRp: NS5 or NS5B) and represents *n* = 125 members of the *Flaviviridae*. The in-frame nucleotide alignment was downloaded from https://talk.ictvonline.org/, adjusted manually, and all positions containing gaps or missing data were eliminated prior to analyses (734 sites in the final dataset). The resulting phylogeny was inferred via the Maximum Likelihood Composite method under the GTR+I+Γ model of nucleotide substitution to account for evolutionary rate differences among sites. The genera *Pegivirus*, *Hepacivirus*, *Pestivirus* and *Flavivirus* are color-coded, in addition to distinct lineages within the genus *Flavivirus*. Statistical robustness of highlighted groupings was determined using the bootstrap approach and values assigned to these branches are percentages derived from 500 replications. Branch lengths are proportional to the scale bar (units: substitutions per site).

The *Flaviviridae* is a diverse group of small enveloped viruses representing over 100 species [10]. Viral genomes from *Flaviviridae* members are non-segmented positive-stranded RNAs ranging from ~9 to ~13 kb in length, and phylogenetic analyses of 125 family members revealed four well-supported genera: *Pestivirus*, *Hepacivirus*, *Pegivirus* and *Flavivirus* (Figure 1 and [10]). Members of the *Flaviviridae* which cause a significant disease burden in humans are highlighted on the tree presented in Figure 1. Genome organization and cytoplasmic replication on endoplasmic reticulum (ER) membranes are conserved between divergent *Flaviviridae* members. However, host-range, tissue-tropism, pathogenicity and modes of transmission can differ greatly [10]. YFV is a member of the *Flavivirus* genus, which is composed of arboviruses exhibiting broad host ranges which can be transmitted to humans from animal reservoirs via insect vectors. This genus contains a number of important human pathogens which can cause acute infections, including dengue virus (DENV), West Nile virus (WNV), Japanese encephalitis virus (JEV) and Zika virus (ZIKV) (Figure 1). Transmission is mediated via mosquito vectors and infection of humans can cause a range of clinical outcomes, ranging from asymptomatic to encephalitis (WNV, JEV and ZIKV) or fatal hemorrhagic fever (YFV and DENV).

YFV continues to pose a very real threat to public health, which has been starkly illustrated by recent outbreaks in Angola in 2015–2016 [1,12,13] and Brazil in 2016–2017 [14,15,16], and associated imports into non-endemic regions [15,17]. This is compounded by a limited vaccine production, which can easily be overwhelmed by a large YFV outbreak, and the collapse of vector control programs [1,14,15], coupled with large populations of unvaccinated individuals in endemic regions and a lack of effective antivirals for treatment of infected patients [18]. Indeed, despite long-standing knowledge of YFVs genomic architecture, favorable growth kinetics in tissue culture and the availability of reverse genetic systems [19,20,21,22,23,24], YFV remains a neglected tropical disease attracting relatively little research attention [25]. This is, in part, due to vaccine availability, which has led to research complacency [3], and recent commentaries have highlighted the need for intensification of YFV research, as many facets of the virus life cycle remain poorly defined [26,27]. These include determination of vertebrate and arthropod host-ranges, identification of co-opted host factors which are essential for YFV propagation in humans and insects, and identification of genetic markers and associated mechanisms underlying differential pathogenesis and observed clinical outcomes in infected humans.

## 2. Clinical Outcomes and Potential Therapeutic Options

Infection with YFV has a broad spectrum of clinical manifestations ranging from entirely asymptomatic to hemorrhagic fever associated with high mortality rates. Indeed, estimates indicate only one symptomatic case for every 7–12 cases that remain clinically silent [5]. In the majority of symptomatic cases, YFV infection is a self-limiting disease with abrupt onset of fever and pain (predominantly myalgia, lumbosacral pain and headache with associated conjunctival injection) that occurs after an incubation period of 3–6 days. In this phase, YFV infection is difficult to diagnose, as symptoms are unspecific. The duration of fever is approximately three days, during which the patient is usually viremic [28]. Laboratory abnormalities during this “period of infection” include elevated liver enzymes, leukopenia and proteinuria. A phase of improvement, lasting up to two days, can be interposed but does not occur in all cases. In around 15% of patients, the disease progresses to a “toxic phase”, which is characterized by the return of fever, nausea, vomiting, epigastric pain and renal insufficiency [18]. This is characterized by systemic viscerotropic disease and liver injury is a hallmark of this phase [29], leading to jaundice and contributing to bleeding diathesis, which may lead to more severe hemorrhaging. It is not well understood why this phase occurs in some patients although host adaptive immune responses are believed to contribute to the severity [5], in addition to underlying genetic factors which predispose Caucasians to a higher mortality rate [30]. The case fatality rate of hospitalized patients ranges between 25% and 50% and is correspondingly higher in South America compared to Africa [31]. Mortality rates may depend on the virulence of the infecting strain, as well as the underlying intrinsic immunity and genetic factors of the exposed population. In addition, cross-immunity to other flaviviruses, such as DENV, is believed to reduce the severity of YFV infections [5,32]. Patients who survive the disease may suffer from fatigue and may have elevated liver enzymes for extended periods of time [29].

Vaccination campaigns represent the only current approach to control and prevent the spread of YFV in endemic regions (see Section 6). Indeed, despite the long history of humankind being afflicted by YFV and the large populations in tropical Africa and Central and South America at risk of potentially catastrophic epidemics, today, treatment options for infected individuals are limited to relief of symptoms and intensive care support of patients with severe disease. Presently, no specific licensed treatment options for YFV-associated severe disease are available. As development of new drugs is expensive and time-consuming, one potential option includes repurposing drugs already licensed for other diseases. This includes drugs targeted at other members of the family *Flaviviridae*, most prominently hepatitis C virus (HCV) and DENV. Ribavirin, a nucleoside analog, in combination with pegylated interferon-α (PEG IFN-α), is effective for the treatment of HCV, a related member of the family *Flaviviridae* (see Figure 1), as well as a variety of other RNA and DNA viruses. In vitro studies and mouse models yielded promising results against YFV, but these results could not be replicated in non-human primates [33]. More recently, considerable efforts have been invested in the development of direct-acting antivirals (DAAs) against HCV and DENV. Sofosbuvir, a DAA licensed for use against HCV, is a nucleotide analog inhibitor of the viral RdRp NS5B. It also has described antiviral activity against the related flaviviruses, ZIKV [34] and DENV [35]. Computer modelling and in vitro experiments in hepatoma cell lines demonstrated sofosbuvir’s inhibitory effects on YFV replication: in a mouse model, sofosbuvir was able to decrease mortality and weight loss associated with liver injury, however, only in the case of pre-exposure treatment [36]. A varying efficacy against YFV has also been demonstrated for other compounds targeting the NS5 RdRp [37], the NS3 helicase [38] and capsid protein [39]. Screening compound libraries via high-throughput in vitro approaches were successful at identifying additional compounds with in-vitro activity against YFV. Patkar et al. (2009) identified two compounds with proposed activity against the NS4B protein [40] and Guo et al. (2016) were able to successfully apply a benzodiazepine compound (BDAA) in a hamster model of YFV infection, where it protected 90% of animals from death via targeting of NS4B [41]. Using RNA interference (RNAi) technology, shRNAs (short hairpin RNAs) targeted at the YFV E and NS1 regions were effective in inhibiting YFV replication in cell lines as well as improving survival in mice when injected intracerebrally prior to exposure to the YFV-17D strain [42]. However, for all the approaches outlined above, demonstration of efficacy in humans or non-human primates remains outstanding.

The host immune response is a believed to contribute to the severity of the “toxic phase”, but approaches targeting the host’s immune response in severe YFV disease are lacking. When patients present with severe symptoms, the therapeutic window of IFNs and other immune modulators may have already passed [43]. The potential benefits of corticosteroids [18] or the therapeutic application of anti-YFV antibodies [44] (see Section 3.1) have not been evaluated but represent promising avenues for further investigation. In summary, many potential candidates for treatment of YFV-induced symptoms have been identified, although none are currently licensed. The toxicity and efficacy of promising anti-YFV therapeutics should be further investigated in animal studies and clinical trials.

## 3. The Replication Cycle of YFV

The replication cycle of YFV is defined by multiple distinct stages, which are shared between divergent members of the *Flaviviridae*. These include (a) attachment of the viral particle to target cells, (b) interaction with surface receptors for internalization, (c) fusion of viral and endosomal membranes and release of the nucleocapsid into cytoplasm, (d) disintegration of the nucleocapsid, (e) translation and replication of the viral genome, (f) assembly and maturation of viral particles and (g) release of nascent virions [45]. Currently, many of these stages are poorly defined for YFV, especially with respect to identification of co-opted host factors, which are essential at every stage of the life cycle. Additionally, how these processes differ between mosquitos and humans requires further investigation.

### 3.1. YFV Virion Composition and E Protein Functions

**Figure 2:**
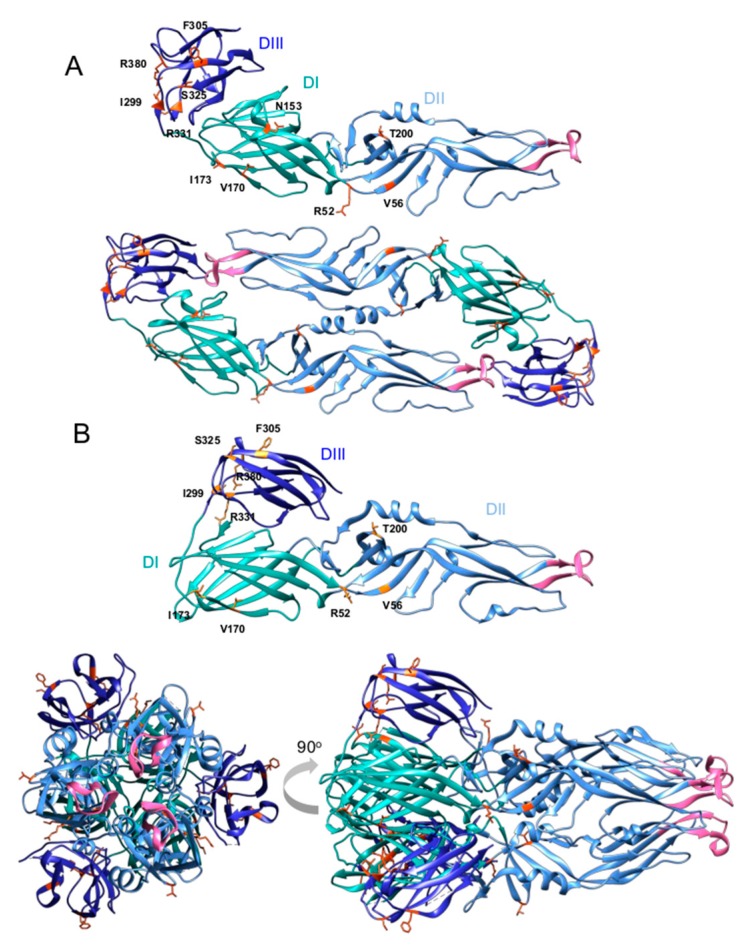
YFV E protein structures with 17D attenuating mutations and fusion peptide highlighted. (**A**) The structure of the YFV-17D E protein pre-fusion monomer is presented with DI, DII and DIII highlighted in different colors. YFV-17D-attenuating mutations are highlighted on the E structure in orange while the fusion peptide is highlighted in pink. Pre-fusion E dimers are presented directly below. (**B**) The structure of the YFV-17D E protein post-fusion monomer is presented with DI, DII and DIII, highlighted in different colors. Again, attenuating mutations are highlighted on the structure in orange while the fusion peptide is highlighted in pink. Post-fusion E trimers are positioned directly below with both top (left) and side (right) views presented. The YFV E structures presented were previously described in [54] (PDB IDs: 6IW1 and 6IW4) and the presented figures were generated in UCSF Chimera [55].

The YFV virion is spherical in shape, with a 40–60 nm diameter [10,46]. The virion is composed of host-hijacked lipid membranes associated with three virally encoded structural proteins. These include the highly basic core protein (C) which binds and encapsidates the YFV RNA genome [47] and two envelope glycoproteins: prM/M (membrane) and E (envelope). The transmembrane domains of prM and E act as membrane anchors and also represent ER retention signals [48]. The E protein of YFV represents a class II fusion protein, which possesses structural homology with the ectodomains of the envelope proteins from divergent arboviruses (related flaviviruses and unrelated alphaviruses), despite no obvious amino acid conservation [49]. The E protein facilitates YFV entry into permissive cells, mediating the interaction between the virus and cell surface receptors, and also elicits host neutralizing antibody responses. YFV E consists of three domains: DI, DII and DIII (see Figure 2A, upper panel). In the pre-fusion state, the E protein forms dimeric structures. DII contains a highly conserved 16 amino acid fusion peptide motif (DRGWGNGCGLFGKGSI) (Figure 2, highlighted pink) [50] and can be found in a parallel orientation to the virion’s lipid envelope in the pre-fusion state (See Figure 2A, lower panel). DIII harbors epitopes essential for neutralization of YFV [51,52]. However, antibody repertoire cloning from YFV patients also identified key neutralizing epitope residues in domains I (residue 71) and II (residues 153-5), which likely combine to form a conformational epitope [53].

The potent anti-E monoclonal antibody 5A identified in [53] was able to neutralize divergent YFV strains (YFV-17D and Angola lineage) [54,56]. The mAb 5A recognizes a conserved region within DII, which is accessible in both the pre- and post-fusion states of the virus. It is suggested that mAb 5A interferes at three stages during YFV infection: (A) by inhibition of virus attachment due to modifying the spatial distance between the E protein and host receptors; (B) by entering early endosomes and inhibiting the “dimer-to-trimer transition” of the E protein by blocking membrane fusion and therefore, blocking the release of nucleocapsid into the cytoplasm and; (C) by binding the E protein trimers in their post-fusion state [54]. Structural and functional studies have suggested conformational change from a dimeric to a trimeric state, which is induced by acidification of endosomes (see Figure 2B) and leads to a release of the nucleocapsid into the cytoplasm via E protein-mediated fusion of virus and endosomal membranes [57,58]. In the post-fusion state, the fusion peptide is located at the apex of the trimer (see Figure 2B, lower panels).

To date, only the structure of the immature YFV particle has been resolved, with the architecture of mature virions containing a processed (pr)M protein still outstanding [59]. By facilitating virus entry into target cells and determining the global structure of the virion, the YFV E is essential for infection. Thus, it represents an ideal target for an intervention at an early state of infection, e.g., using a structure-based drug design or E-targeted monoclonal antibody (mAb) therapies [44]. Antiviral mAbs represent viable therapeutic options, including palivizumab (marketed as Synagis), which is licensed for use against RSV in humans [60], and a number of anti-HIV mAbs in clinical trials [61,62]. In most cases, therapeutic mAbs interfere with virus entry via targeting the envelope glycoprotein(s), thus preventing further dissemination. However, currently, while mAb based therapies have proven effective, they are also expensive, which limits their widespread application in the resource constrained settings where YFV epidemics often occur.

### 3.2. YFV Entry: Implicated Host Factors

Out of the three domains of the YFV E protein, DIII is suspected to mediate interaction with cellular receptors during infection [45]. While flavivirus entry into target cells is incompletely defined, many host molecules have been implicated in the process. These include CD209 and the mannose receptor for DENV entry [63,64] and G protein-coupled receptor kinase 6 (GRK6) for WNV entry [65]. The related GRK2 was reported to both promote YFV entry and enhance viral RNA synthesis, properties which were conserved across key members of the *Flaviviridae*, including DENV and HCV [66]. The transmembrane immunoglobulin and mucin domain (TIM) and TAM (Tyro3, Axl and Mer) families of phosphatidylserine receptors have been shown to mediate DENV infection, with TIM-1 and TIM-4 also enhancing infection rates for WNV and YFV-17D to differing degrees, while herpes simplex virus-1 (HSV-1) infection rates were unaffected [67]. Therefore, these cellular factors represent general enhancers of flavivirus infection. Additionally, candidates implicated in YFV host-cell attachment include heparin sulfate, a member of the glycosaminoglycan (GAG) family, which also facilitates attachment for the related flaviviruses DENV [68] and tick-borne encephalitis virus (TBEV) [69], as well as unrelated viruses, including HSV and human immunodeficiency virus (HIV) [70,71]. Indeed, the antiviral effect of PI-88, a heparin sulfate mimetic, was demonstrated against DENV and neurotropic flaviviruses [72]. Despite reports of multiple factors which are potentially involved in YFV entry, it is still unknown whether a ubiquitous cellular receptor is responsible for facilitating flavivirus infection of permissive cells [73]. Similarly to DENV, YFV is able to infect a range of cell-types from diverse host species, indicating either binding to a ubiquitous cell-surface receptor, or the ability to engage multiple distinct receptors [74]. RNAi screening has revealed many potential host factors influencing WNV or DENV infection of human or drosophila cell-lines [65,75]. Analogous to mammalian CD209, an *Aedes aegypti* C-type lectin, mosGCTL-7, has been described as an entry factor for JEV [76]. Previous studies already identified mosGCTL-1 and mosPTP-1 as cascade receptors facilitating flaviviral entry in mosquitoes [77]. Thus, distinct flaviviruses may use conserved entry factor(s) for entry into permissive cells in mosquito vector species.

Divergent entry mechanisms have been observed for even closely related YFV strains. The parental Asibi strain and the derived YFV-17D vaccine strain both infect permissive cells by a pH-dependent fusion mechanism, although these related viruses enter via distinct routes [78]. Both strains enter in a dynamin-2 dependent fashion and traffic through Rab5- and Rab11-positive early and recycling endosomes. However, the wild-type Asibi strain enters via clathrin-mediated endocytosis, while YFV-17D utilizes a clathrin-independent route. YFV-17D also exhibits enhanced binding and entering capabilities in vitro, and initiates inflated antiviral responses when compared to its wild-type progenitor [78]. These divergent properties can be pin-pointed to eleven mutations within the E protein’s primary amino acid sequence (see Figure 2), which occurred during long-term passaging of the Asibi strain in mouse brains and chicken embryos to generate the attenuated YFV-17D derivate (see Section 6) [20]. Of note, a T380R mutation in the present E-DIII vaccine strains creates a RGD motif, a known integrin binding site [78,79,80], and is part of a heparin sulfate binding domain: increased heparin sulfate affinity is known to be important for viral spread and attenuation [68,78]. In summary, mechanistic insights into the YFV infection process, in addition to many host factors implicated in YFV entry, have been described. Nevertheless, identification of *bona fide* receptor(s) integral for YFV entry remains outstanding. The entry cascade of YFV is comprised of multiple steps which could be targeted by therapeutic intervention to inhibit further virus spreading within infected patients. For example, targeting the HCV entry factor CLDN1 via a mAb inhibited viral entry and promoted viral clearance in a humanized mouse model [81].

### 3.3. Polyprotein Processing, RNA Replication and Virion Maturation

**Figure 3:**
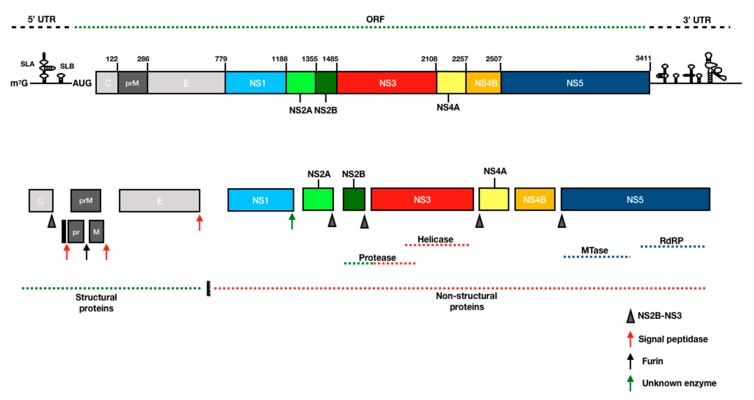
Schematic representation of the YFV genome and encoded polyprotein. The genome of YFV is a single-stranded positive-stranded RNA of approximately 11kb encoding a single ORF containing three structural proteins (C, prM, and E) and seven nonstructural proteins (NS1, NS2A, NS2B, NS3, NS4A, NS4B, and NS5). The genome has methylated type 1 cap at the 5′ end and is not polyadenylated. Secondary structures (stem-loop A and B; SLA/SLB) in the 5′ and 3′ UTR are required for translation and RNA synthesis. The polyprotein is post-translationally modified by viral and host proteases into 10 mature proteins which are incorporated into the ER membrane.

After entry and uncoating, viral RNA is transported to the endoplasmic reticulum (ER) and translated as a single polyprotein precursor. Host factors RPLP1 and RPLP2, phosphoproteins that bind to ribosomes, are essential for efficient ribosomal translation of the YFV polyprotein and have panflaviviral activity [82]. The YFV polyprotein is post-translationally modified by cellular glycosyltransferases, and the liberation of ten mature viral proteins is mediated by both host and viral proteases [83,84]. The processed polyprotein produces three structural proteins which comprise the virion (see above) and seven nonstructural (NS) proteins (NS1, NS2A, NS2B, NS3, NS4A, NS4B, and NS5) [85] which are involved in viral RNA replication on ER membranes (Figure 3 and Table S1). After translation of the genome, a domain of the RdRp NS5 binds to a promotor sequence on the stem-loop A (SLA) at 5’ end of 5’ untranslated region (UTR) to promote the synthesis of uncapped negative-sense RNA, which is produced at low levels from the positive-sense RNA template [86,87]. Flavivirus positive-stranded RNA genomes are similar to vertebrate mRNAs, although they differ in critical aspects: they possess a methylated type 1 cap structure at the 5′-terminus (me^7^-GpppA-me^2^) and lack a polyadenylation signal at the 3′-terminus. The type 1 cap is synthesized by virally encoded proteins: an NS3-encoded RNA triphosphatase [88] and the two NS5-encoded enzymes: methyltransferase [89,90] and guanylyltransferase [91]. NS3 and NS5 possess additional functions and constitute the core of the flaviviral RNA replication complex [85] which occurs on ER membranes [92]. Indeed, DENV NS3 and NS5 were identified on the virus-induced membrane vesicles of the ER, which are open to the cytoplasm [93]. A serine protease encoded at the N-terminus of NS3 is involved in cleavage of multiple NS protein junctions [94] and requires the NS2B cofactor for its activity [95]. NS3 also possesses a C-terminally encoded NTPase/RNA helicase to remove viral RNA secondary structures and double-strand RNA (dsRNA) intermediates, generated by NS5 during virus replication [96]. The flavivirus NS5 protein encodes the viral RdRp, synthesizing negative-stranded RNA, which forms a double-stranded complex with the genomic template [97]. Experiments with DENV demonstrated that the phosphorylation of NS5 controls the interaction with NS3 [98]. Furthermore, mutation of the phosphorylation site on YFV NS5 (S56) disrupted the capping process via inhibition of the methyltransferase activity and inhibited viral replication [99]. NS1, NS2A, NS4A and NS4B represent hydrophobic membrane proteins which modify the structure of the ER, creating a scaffold that anchors the replicase complex during virus assembly, although their precise functions remain unclear [100,101,102,103,104,105,106].

Flaviviral replication is coupled with the process of assembly [107]. Open pores on the lumen of virus-induced ER membrane vesicles allow spatial coordination between the replication and assembly processes [108], facilitating association of newly synthesized viral RNA with structural proteins to produce immature virions during virion assembly [109,110]. The viral lipid-envelope is acquired from the host cell by budding from the lumen of the ER [111] and virion maturation occurs through trans-Golgi network trafficking to the cellular surface, where furin cleavage of the pr peptide from the M protein produces a rearrangement of the E protein [112], generating infectious virions that are fusogenic under low pH conditions [27]. Viral replication, assembly, and maturation, as well as host factors essential for viral propagation, are potential targets for the development of anti-YFV drugs. However, a better understanding of determinants of viral replication and the virus-host interactome are required for YFV: many of the critical steps of the flavivirus replication cycle are described for DENV or WNV, but confirmation with YFV is lacking.

## 4. Host Responses to YFV Infection

### 4.1. Cytokine Induction by YFV

Using paraffin-embedded liver tissue from patients who succumbed to fatal YFV disease, it was demonstrated that CD4^+^ T cells represent the main infiltrating cell population into liver tissue, with a high proportion of cells expressing TGF-β and, to a lesser extent, TNF-α and IFN-γ [113]. YFV-induced apoptosis markers were prevalent in infected hepatocytes, indicating that apoptosis, rather than necrosis, is the primary cell-death mechanism in YFV-infected livers [113]. With respect to serum markers, higher concentrations of IL-6, MCP-1, IP-10, TNF-α, and IL-1RA were detected in human sera from YFV-infected patients with fatal outcomes when compared to sera from non-fatal outcomes [114]. Vaccination with YFV-17D also increased the production of TNF-α, IL-1RA and IL-6 in plasma at 2 and 7 days post-vaccination [115]. Together, these results indicate that induction of pro- and anti-inflammatory cytokines is associated with the immunopathology of YFV infection and responses to YFV-17D vaccination.

### 4.2. Cellular Responses to YFV

Dendritic cells (DCs) are essential antigen-presenting cells, recognizing pathogens by toll-like receptors (TLRs) to trigger maturation and the induction of adaptive immunity and immunological memory. The activation of DCs by YFV-17D through TLR 2, 7, 8 and 9 was shown to induce a broad range of innate and adaptive responses [116]. Activated plasmacytoid dendritic cells (pDC) rapidly secrete large amounts of type I-IFNs to modulate effector molecules and cell populations of the innate and adaptive immune system. YFV also was shown to induce the production of IFN-γ and type III-IFNs from pDCs via interaction with TLR7 or cell contact, and immature virions were more efficient at stimulating pDCs than mature virions [117]. However, YFV also possesses the ability to antagonize the IFN system. YFV NS5 interacts with STAT2 when STAT1 is phosphorylated during type I IFN signaling: the E3 ubiquitin ligase TRIM23 polyubiquinates NS5, mediating STAT2 binding and subsequent inhibition of IFN signaling [118]. Human and rhesus macaque monocyte-derived dendritic cells (MoDC) and macrophages (MDM) were also shown to be susceptible to infection with YFV wild-type Asibi and 17D vaccine strains, with differences in cytokine responses to infection observed between strains. Additionally, only MoDCs infected with YFV-17D could initiate IFN-γ and IL-2 expression in CD4^+^ T cells, confirming differential patterns of immune induction between attenuated and wild-type YFV [119]. Natural killer (NK) cells represent an essential component of innate immune responses, shaping adaptive and antiviral immunity [120]. NK cells were shown to be the highest producers of IFN-γ, which protects against viscerotropic disease in YFV-17D infection but not for wild-type virus, indicating that YFV vaccine attenuation results in increased sensitivity to IFN-γ [121]. Replication of YFV-17D in NK cells was enhanced by STAT1 knockout, although NK cells do not appear to be a major reservoir for YFV replication [122]. After YFV-17D vaccination, NK expression of TLR3, TLR9 and CD69 increases, concomitant with increased circulating IFN-γ [123] and 17D-YFV infection stimulated vigorous early NK cell responses in vivo primed by type I and III IFNs [124].

### 4.3. Pattern Recognition Receptors and YFV

In addition to TLRs, the pattern recognition receptors (PRRs) RIG-I, MDA5 and LGP2, which form part of the RIG-I like receptor (RLR) family, are critical for initiating the IFN response to infection via recognition of intracellular viral RNA [125]. While RIG-I recognizes dsRNA, which possesses a triphosphate and U or A-rich motifs, MDA5 preferentially recognizes long dsRNAs, [126]. Both RIG-1 and MDA5 have important roles in restricting flavivirus infections ([127] as reported for DENV [128], WNV [129] and JEV [130]), interacting with both MAVS and STING to mediate their antiviral effects [131]. However, viral genome modifications can prevent RIG-I recognition, with cap1 2′O-methylation of YFV RNA constituting an RIG-I evasion mechanism [132]. Cyclic GMP-AMP synthetase (cGAS) is a cytoplasmic DNA sensor, synthesizing, 2′3′-cGAMP from ATP and GTP to mediate the activation of STING. After TBK1-mediated phosphorylation of IRF3, nuclear translocation of IRF3 and NF-κB induces type I-IFN and cytokine expression [133]. In addition to cytoplasmic DNA, cGAS has also been implicated in the recognition of RNA viruses, including flaviviruses: cGAS knockout mice were more susceptible to lethal WNV infection outcomes when compared to wild-type mice [134], and DENV is able to target cGAS for degradation via NS2B, inhibiting IFN production [135,136]. YFV NS4B exhibits strong homology with the C-terminus of STING and can inhibit its activity [137]. However, while human STING can be targeted for cleavage by ZIKV, DENV, WNV and JEV proteases, it is not susceptible to cleavage by either YFV-17D or YFV-Asibi proteases [138]. Finally, nucleotide-binding and oligomerization domain (NOD) like receptors (NLRs), are cytoplasmic receptors that trigger the engagement of inflammasomes and signalosomes after the detection of viral danger signals. This occurs via activation of caspase-1, which mediates maturation of pro-inflammatory cytokines (e.g., IL-1β and IL-18) and secretion of the alarmin HMGB1 [139]. While inflammasome activation has been reported for WNV and DENV infection [126], the exact danger signals detected and its significance in YFV infection remains unclear. Together these studies implicate RLRs and the cGAS-STING axis as important modulators of YFV infection, although further investigation is required. Additionally, whether inflammasome activation is mediated by YFV replication warrants further study.

### 4.4. Anti-YFV Effectors

The mammalian chaperone protein DNAJC14 was demonstrated to confer protection from YFV-mediated cell-death via inhibition of YFV replication and possesses broad activity against diverse *Flaviviridae* members [140]. This factor is constitutively expressed and not IFN-inducible. The breadth and antiviral potency of over 380 human interferon-stimulated genes (ISGs) were tested against a panel of important pathogenic viruses, including YFV, using lentiviral overexpression systems in different cellular backgrounds [141]. This comprehensive study identified antiviral effectors which inhibited YFV replication, including IRF1, TREX1, IFITM3, RTP4, IFI6 and HPSE: in contrast, the IFN regulated genes APOBEC3A, FAM46C, IDO1, LY6E, MCOLN2 and ADAR genes enhanced YFV replication [141]. More recently, CRISPR/Cas9 knockout genetic screening confirmed IFI6 as an IFN-induced gene with anti-YFV activity and provided mechanistic insight into its mode of action [142]. IFI6 has broad activity against *Flaviviridae* members but minimal activity against other viruses, interfering with flaviviral proteins when they invaginate ER membranes through interaction with the ER-resident heat shock protein 70 chaperone BiP [142]. Together, these results point to the complexity of the IFN system: expression of both pro- and anti-YFV effector genes are modulated by the type I IFN response. In addition, some genes have panflaviviral potency, while others are virus specific. The contribution of patient and tissue-specific differences in ISG expression to viral clearance and the correlation between ISG induction and differential disease outcomes is currently unclear.

## 5. Tropism of YFV

In humans, after transcutaneous inoculation, YFV exhibits broad patterns of tissue tropism and is disseminated systemically via infection of multiple white cell subsets. With respect to organ tropism, the liver is the primary target, although the heart, kidneys, liver, spleen, and less frequently, the brain tissue, can support YFV replication [18]. The natural host range of YFV includes humans, non-human primates and mosquitos. Additionally, wild animals from the diverse orders Artiodactyla, Carnivora and the superorder Xenarthra have been reported to be serologically positive for anti-YFV antibodies, and the elicitation of antibody responses against YFV indicates a potentially productive infection within these species, indicating that diverse mammalian species could represent YFV potential reservoirs [143] (see Section 8.3).

### Mouse Models for YFV

YFV research is currently limited as it remains challenging and expensive to establish workflows with non-human primates. While YFV has a limited host species-tropism, artificial infection of laboratory mice and hamsters is possible, although the course of disease differs from that observed in humans [144]. Nevertheless, a number of studies in genetically modified or humanized mice have yielded insights into YFV infection and dissemination in vivo. Single cell-tracking of YFV-17D dissemination in different mouse models identified novel YFV replication reservoirs in secondary lymphoid compartments, with hematopoietic STAT1 knockout increasing virus-induced pathogenicity [122]. Double-humanized mice have been developed, possessing both human immune systems and hepatocytes [145,146], and these animals represent ideal tools for monitoring systemic YFV infection in vivo as both compartments are permissive for YFV. In addition to hepatotropism, YFV can also be neurotropic in rodents, primates and humans [147,148]. In contrast to polio virus and reovirus, which infect the brain via the CNS, YFV-17D infects brain tissue by crossing the blood-brain barrier [149]. A mouse-neurovirulent strain (SPYF) based on the Porterfield 17D (PYF17D) strain was derived by additional passaging in SCID mice, exhibiting enhanced neurovirulence and neuroinvasiveness. This enhanced neurotopism was correlated with 13 amino acid substitutions, five of which were located in the E protein [80]. Arboviruses can encounter dissemination barriers in mosquitos, restricting the spreading of the virus within the vector species. These include innate immunity related pathways as well as physical tissue barriers associated with the midgut and salivary glands [150,151]. However, while patterns of YFV-17D dissemination in immunocompetent mice were restricted, infection of *IFNAR* knockout mice resulted in broad dissemination preceding viral clearance, indicating that no barriers to dissemination exist in the absence of type I IFN [152]. Furthermore, while I and II type IFNs contribute to control of YFV-17D infection, type III IFN was demonstrated to have immunomodulatory functions and was protective against YFV-17D neuroinvasion in mouse models [153]. YFD-17D infection of IFN-α/β receptor and STAT1 signaling molecule deficient mice resulted in systemic viscerotropic infection: a course of disease which resembles that observed in humans rather than the typical encephalitic disease course commonly reported for mice [154]. Finally, while many flaviviruses share the common ability to antagonize IFN signaling and therefore enhance viral replication, the YFV NS5 is unable to bind murine STAT2 and in contrast to ZIKV and DENV, is unable to engage human STAT2 in murine cells. Murine STAT2 was demonstrated to repress YFV replication in murine cells, which was correlated with an absence of IFN-α/β-mediated NS5 ubiquitination [155]. Together, these mouse studies enhance our understanding of YFV dissemination and host control in vivo. These findings are useful for the development of the next generation of small animal disease models for YFV, which should endeavor to recapitulate the disease course seen in humans. Thus, the generation of an immunocompetent mouse model would be beneficial, which could be realized by knocking out downstream specific anti-YFV effectors that directly inhibit differential aspects of the YFV lifecycle, rather than upstream master-regulators of the antiviral response. Screening approaches could identify such factors.

## 6. YFV Vaccine: Development and Limitations

Immunization is one of the most successful and cost-effective health interventions. Over the past decades, immunization has achieved eradication of smallpox, lowered the global incidence of polio by 99% and reduced illness, disability and death from vaccine-preventable diseases [156]. At present, there are eleven live attenuated vaccines (LAV) and among them, the YFV vaccine is the oldest and the most potent. As there are no specific treatments for YFV infection, vaccination represents the only current protection against the disease.

### 6.1. History and Development of the YFV Vaccine

In the 1920s, two parallel approaches were taken to develop a live attenuated vaccine against YFV. In 1927, YFV was isolated from a Ghanaian patient suffering from mild yellow fever and inoculated into rhesus macaques by direct blood/serum transfer [157]. Max Theiler and colleagues discovered that this “Asibi” strain, which was refractory for growth in other small laboratory animals, was able to replicate in the brains of mice following intracranial injection. Following sequential passage in mouse brains, reduced viscerotropic virulence was observed, coupled with enhanced neurotropic properties. Therefore, the Asibi strain subsequently underwent serial passage in chicken embryo tissue, after head and spinal column removal. After 176 passages, the attenuated YFV-17D strain had lost its viscerotropism, neurotropism and mosquito competence, while retaining its immunogenicity [8]. Concurrently, French researchers isolated YFV from a patient in Dakar, followed by serial passage in mouse brains. This attenuated strain, termed the French neurotropic vaccine (FNV), was isolated at passage 260 and was widely used in the francophone countries of Africa, virtually eradicating the disease. However, serial passaging in mouse brain increased FNV’s neurotropism, leading to high levels of post-vaccinal encephalitis in children. Consequently, the use of FNV was discontinued in the 1980s [158]. The 17D subculture is the seed strain for all modern-day YFV vaccines. There are three sub-strains obtained from the original 17D vaccine: the 17DD (Passage 195), 17D-204 (Passage 204) and the 17D-213, derived from 17D-204 and used by the Robert Koch Institute, Germany. These strains have slightly different genomic sequences: in particular, glycosylation sites on the envelope protein vary, although no differences in attenuation or immunogenicity have been observed [159]. The original passage 176 virus no longer exists, and comparisons are mainly made between the three strains outlined above [160].

The Centre for Disease Control and Prevention (CDC) recommends YF vaccination for travelers to- and residents of- endemic areas aged 9 months and older. Vaccination is also recommended for people who work in research laboratories and may be exposed through needle stick injury or inhalation of aerosolized viral droplets [161]. The YFV vaccine is a freeze-dried live-attenuated virus delivered subcutaneously. It is available as a single dose or multi-dose vials to be stored at 2–8 °C. It is reconstituted with normal saline and should be used within an hour of reconstitution [162]. Usually, a single dose confers life-long immunity, but a booster is recommended every 10 years for people at risk of exposure or people who were infected with HIV when they received their last dose. Additional doses can also be administered to people who receive hematopoietic stem cell transplants after receiving the vaccine and who are immunocompetent [163].

### 6.2. Immune Responses Post Vaccination

When humans are vaccinated with YFV-17D, anti-yellow fever serum is detected as early as 2 days post immunization [164,165]. It induces low-grade viremia in half of the vaccines and elicits protective neutralizing antibody (nabs) levels in 99% patients [166]. Nabs represent the main correlates of protection induced by YFV-17D. These are quantified using plaque reduction neutralization tests (PRNT) demonstrating robust responses as early as 6 days following vaccination and peaking in titer after 30 days, suggesting a functional memory response [166,167]. While the humoral arm of vaccine-induced immunity has been studied in detail, a role for T cells is expected in the development of nabs [26,168], and strong initial activation of innate immunity primes subsequent adaptive responses [169,170,171]. The role of other cell types, including NK cells and monocytes, has been inferred in generating protective responses post YFV-17D vaccination. A role for TLRs in NK cell activation was identified in YFV-17D-induced immune responses and may contribute to protective immunological memory [172]. Indeed, a systems biology approach indicates a coordinated induction of transcription factors precedes a broad persistent immune response that incorporates all effector cells of the immune system [173].

### 6.3. Success Story

The 17D vaccine has been in use for over 75 years. Over 600 million doses have been administered to date, although rare cases of serious adverse reactions are described [162]. Remarkably, very few cases of vaccine failure have been documented due to its near-global sero-conversion rate in human vaccinees and induction of persistent immunity [174]. Due to its success, the YF vector backbone is currently being trialed as an antigen delivery platform for other experimental vaccines including HIV-1, Lassa fever, malaria and DENV [175,176,177,178,179,180]. After early successes in outbreak prevention and control in the mid-20th century, largely due to mass vaccination campaigns and routine child immunization programs in endemic countries, a reduction of YFV cases was observed globally [181]. However, when Angola was hit by urban outbreak which spread to neighboring countries, including in the Democratic Republic of the Congo’s capital Kinshasa, the epidemic created an urgent need for more than 28 million doses of the YFV vaccine, exhausting the existing global vaccine supply [182,183,184]. Furthermore, an unusually large outbreak of the disease in Brazil in early 2017, including areas that previously were not considered endemic [185,186], confirmed the need to review the previous strategies [185]. In order to maximize the impact of limited vaccine supplies in a context of depleted global stockpiles, fractional doses of YFV vaccine were administered. Because promising data were available on the immunogenicity of fractional dosing, 10 million individuals were immunized with 2 million doses of 17DD [183,186].

### 6.4. Limitations: Production and Adverse Reactions

The disease has re-emerged as a public health threat in recent decades, triggered by different factors, such as climate change, increasing population movement and expanding ranges of mosquito vectors. It is estimated that vaccine manufactures can produce a combined 80 million doses per year [160]. However, as YFV-17D-based vaccines are still produced in chicken eggs, this results in issues with supply and demand during outbreaks due to limited production capabilities [187]. Another limiting issue during current production is the stability of live-attenuated vaccines. Antigen instability is an inherent attribute of vaccines, compounded by a requirement for conformationally correct three-dimensional structures that elicit protective host responses to these complex bio-therapeutics. Therefore, more stable vaccines with reduced dependency on the cold chain represent an area for future vaccine development [188]. Thermal stability is important for the manufacture, distribution and administration of vaccines, especially in tropical developing countries where adverse field conditions exist. Current live-attenuated vaccines exhibit relatively poor liquid stability in clinical settings, and clinicians are instructed to discard the YFV-17D vaccine one hour after reconstitution [189]. This issue can be best addressed by stabilization. Indeed, poliovirus has been experimentally stabilized by the addition of pirodavir and deuterium oxide, with viral RNA and viral capsid resisting a 10-h incubation at 42 °C [190].

Despite the protective nature of the YFV-17D vaccine against potentially fatal hemorrhagic fever, there are certain contraindications, including children aged less than 9 months or 6 months during an epidemic, pregnant women, lactating women, people with severe immunodeficiency due to symptomatic HIV or the presence of thymus disorder, and hypersensitivity to chicken eggs [161,187]. In addition, there are common side effects of the vaccine, ranging from muscle aches, chills, flu-like symptoms, joint pain, mild fever, mild rash, muscle weakness and swelling at the injection site. Using transcriptomic and metabolic profiling of 17D-204 vaccinees, it has recently been demonstrated that symptomatic outcomes to vaccination were associated with baseline endoplasmic reticulum stress and reduced tricarboxcylic acid cycle activity, initiating downstream proinflammatory responses [191]. The use of a live attenuated vaccine can prevent yellow fever, but vaccine-associated neurologic disease has been reported and is a safety concern [192,193,194]. In severe cases, memory loss, problems with breathing, behavioral changes, seizures, viscerotropic disease, encephalitis and death can occur. In addition, the vaccine is known to interact with about 42 drugs, but overall, the risk of developing adverse effects from vaccination is low. The causes of serious adverse events to vaccination were previously unknown. However, recent studies implicated abnormal immune responses and identified patient-specific polymorphisms in immune-associated gene loci, including *CCR5*, *RANTES* and *IFNAR1* [193,195,196,197]. Finally, the risk of a large-scale epidemic in Asia remains daunting and YFV is the only infectious disease where an international vaccination certificate is required [160]. However, not all countries enforce international health regulations with respect to requiring a vaccination certificate for entry, and there has been evidence of fake/forged certificates [198].

### 6.5. Future Directions

Yellow fever is a re-emerging infectious disease, as vector control and routine immunization strategies have dwindled in recent years. To combat this, the WHO has initiated a global strategy to eliminate yellow fever epidemics (EYE) by 2026, focusing on management of global vaccine supply, including emergency stockpiling, improving surveillance and outbreak control [199]. Accordingly, 1.3 billion individuals will be vaccinated in the next five years, focusing on endemic areas and areas where YF is sporadic [199]. To guide responses to future outbreaks, mathematical modelling studies are being used to evaluate the efficacy and effectiveness of full and fractional dosing regimens. The model explores actual and hypothetical vaccination strategies and the impacts of possible human reactive behaviors [200,201,202].

To overcome production limitations and reduce adverse reactions, alternative vaccination approaches should be explored, including vaccinia vector-based systems, DNA vaccines, inactivated 17D vaccines and recombinant subunit vaccines produced in both mammalian cells and plants [203,204,205,206,207,208]. In line with this, current efforts to develop a new inactivated YF vaccine to reduce serious adverse events have shown good immunogenicity in preclinical and clinical studies [209,210,211]. In addition, DNA-launched vaccine technology has the advantages of both traditional DNA and live-attenuated vaccines. This platform minimizes the potential for reversions or adverse effects of traditional live-attenuated vaccine, ensures genetic stability, and results in efficient immunization in animal models [212]. The use of live attenuated viruses can lead to reversion, generation of new biological properties and recombination [213,214]. Therefore, as an alternate to conventional attenuation approaches, codon re-encoding was developed with its basic principal to introduce synonymous mutations into the viral genome [215] that would impose the least detrimental effects on viral fitness [216]. This approach has been applied to other RNA viruses, including chikungunya (CHIKV) and TBEV [217,218].

In summary, in addition to vaccination, vector control is imperative: one should also consider therapeutic options and not just rely on prophylactic vaccines. Fractional dosing for other strains needs to be validated for future outbreaks, and there is a need to manage the potential risk of YFV introduction to Asia. In the coming decade, vaccine manufacturers are expected to meet the global demand of 1.38 billion doses needed to eliminate the risk of YF epidemics [199]. Hence, novel manufacturing techniques to produce large quantities of yellow fever vaccine at short notice are required which also eliminate the chicken embryo proteins that lead to allergic reactions. The mechanism by which YFV-17D mediates protection against the virus is not well defined. This is due, in part, to the limited animal models available to evaluate vaccine-induced immune responses [168] and the difficulty accessing samples from fatal YF patients. Together, this highlights the need for further research and refining of this effective vaccine [183].

## 7. Global Diversity of YFV

**Figure 4:**
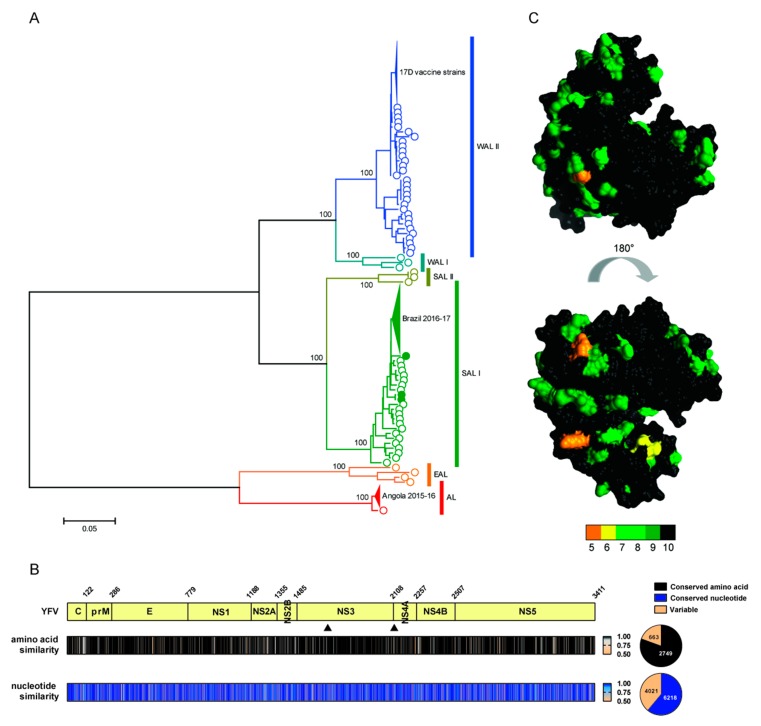
YFV genomic and amino acid diversity. (**A**) Phylogenetic analysis of YFV genomes. The presented tree was generated using PhyML [220] from full-length ORF sequences. Nucleotide sequence alignment (10239 nucleotides) was generated in MEGA7 [11] and adjusted manually to ensure maintenance of the polyprotein ORF. Collapsed clades in the YFV phylogeny contain closely related isolates from the 2016–2017 Brazilian outbreak (*n* = 52, green triangle), the 2015–2016 Angolan outbreak (*n* = 16, red triangle) and 17D vaccine strains (*n* = 46, blue triangle). The green filled circles in SAL I (*n* = 3) represent YFV sequences isolated from non-human primates during the 2016–2017 Brazilian epidemic, which co-cluster with previously sampled non-outbreak sequences and are antecedent to the main outbreak grouping (green triangle). The statistical robustness of YFV lineages were calculated using the bootstrap approach in MEGA7 [11] and values assigned to the branches are percentages derived from 500 replications. Branch lengths are proportional to the scale bar (units: substitutions per site). (**B**) Conservation and variability in YFV genomes. Amino acid and underlying nucleotide similarity plots for globally sampled YFV genomes are presented. For the purpose of positional referencing, a cartoon of the YFV reference strain polyprotein is provided directly above, with coordinates of polyprotein cleavage sites for the liberation of mature proteins highlighted. The triangles positioned below indicate the position of the NS3-encoded RNA helicase. Degrees of amino acid or underlying nucleotide or similarity at individual sites/residues in global alignments were calculated in CLC Genomics Workbench (Qiagen Aarhaus) and relative similarities are color-coded according to the scale bars positioned to the right. The associated pie charts indicate the proportion of conserved or variable sites in each dataset. The numbers inset indicate the actual numbers of variable and conserved amino acids or nucleotides in the alignment. (**C**) Conservation and variability in the YFV RNA helicase. Amino acid conservation and variability estimated in (**B**) were mapped onto the YFV NS3 RNA helicase structure. Relative conservation of amino acids on the helicase structure are color-coded according to the scale bar positioned below. Completely conserved residues are colored black while variable residues highlighted dark green through to orange. The YFV helicase structure presented was described in [221] (PDB ID: 1YKS; 440 amino acids) and the conservation and variability were visualized on the structure using UCSF Chimera [222].

At the time of writing this review, *n* = 1112 YFV sequences were deposited on the NCBI GenBank sequence database. To assess patterns of globally sampled YFV diversity, we performed phylogenetic analyses of all available YFV full-length ORF sequences (*n* = 178) (Figure 4a). Collapsed clades contain closely related isolates from recent Brazilian and Angolan outbreaks, in addition to 17D vaccine strains. Based on this analysis, global YFV sequences can be divided into six well-supported lineages: West African lineages I and II (WAL I and WAL II), South American lineages I and II (SAL I and SAL II), the East African lineage (EAL) and the Angola lineage (AL). In agreement with a previous phylogenetic analysis of partial [219] and full-length ORFs [16,27], our expanded analysis illustrates that South American lineages are monophyletic, sharing a common ancestor with the West African lineages, and confirms a deep-divergence between these lineages and the more distantly related East African and Angolan strains, indicating an ancient separation. This observation is consistent with an African origin for YFV, with South American lineages representing historical import of West African YFV strains linked to European colonial expansions and the slave trade [2,6,18,219].

While clinical YFV isolates can be readily propagated in tissue culture, the majority of studies utilize the YFV-17D vaccine strain which has atypical properties [78], or its wild-type progenitor, the Asibi strain, representing only a small fraction of the global YFV diversity (Figure 4a). Consequently, the phenotypic correlates of YFV global sequence diversity are incompletely defined and future studies should address this discrepancy: it is currently unknown whether infection with isolates from different YFV lineages contributes to differential pathogenicity or mortality rates in humans. However, in spite of these deep-divergences apparent in the YFV phylogeny, the 17D vaccine provides robust protection against strains from all known YFV lineages.

### YFV Genome and Polyprotein Conservation

To visualize variability across YFV genomes and encoded polyproteins, similarity plots were generated from YFV nucleotide and amino acid alignments. Inspection of genetic and amino acid variability in globally sampled YFV isolates highlights conserved and variable regions in the genome and encoded polyprotein (Figure 4b). Of note, YFV nucleotide variability is much greater than amino acid variability, indicating strong purifying selection shapes global patterns of YFV evolution. This is likely a reflection of the selective constraints imposed by the viral transmission cycle: YFV experiences sequential bottlenecks upon continued cycling through an insect vector, coupled with requirements for host factor compatibility in both vertebrates and arthropods. Furthermore, the YFV RdRp has a relatively high fidelity for an RNA virus, with an estimated mutation rate per site of 1.9–2.3 × 10^−7^ [223]. Together, these analyses indicate that both the structural and non-structural proteins of YFV exhibit extensive conserved regions which are therefore viable targets for pharmacological intervention. Consequently, the development or repurposing of direct-acting antiviral compounds targeting YFV proteins should represent an achievable goal. Indeed, it was previously noted that the RNA helicase domain of NS3, integral for RNA duplex unwinding during viral replication, has a conserved function in the *Flaviviridae* and represents a potential drug target [224]. Mapping of global amino acid diversity on to the resolved helicase structures for YFV [221] is presented in Figure 4c, highlighting the conserved regions of this essential virtual protein. Together these analyses, conducted using sequence and structural data available in public databases, can inform rational development of antivirals targeting different components of the YFV virion or replication machinery. Currently, structural data is only available for YFV E, NS3 and NS5 proteins. The elucidation of structural information for the outstanding YFV proteins will expand the number of potential viral targets for future structure-based antiviral drug design studies.

## 8. Monitoring YFV Outbreaks, Disease-Course and Reservoir Species with NGS

### 8.1. Diagnostic Utility of NGS

The use of NGS technologies allows recovery of complete viral genomic sequences directly from infected patients’ material, and these metagenomic approaches have revolutionized infectious disease outbreak diagnostics and surveillance in recent years [225]. In Africa and South America, YFV co-circulates with multiple endemic VHFs which can emerge in humans sporadically, complicating initial diagnosis. These include Ebola viruses (EBOV), Marburg virus (MARV), Rift Valley fever virus (RVFV), Junin virus (JUNV) and Crimean-Congo hemorrhagic fever (CCHFV). The diagnostic capacity of NGS to discriminate between hemorrhagic fever viruses from diverse families and orders, including the *Arenaviridae*, *Bunyavirales*, *Filoviridae* and *Flaviviridae*, has been reported [226]. Indeed, using 454 sequencing (Roche), YFV was identified as the causative agent of an outbreak of VHF in Northern Uganda in 2010 [227], validating the diagnostic utility of NGS technologies for discriminating YFV outbreaks from unrelated hemorrhagic fever viruses.

### 8.2. Outbreak Monitoring in Real-Time

More recently, as NGS technology has advanced, hand-held nanopore sequencing devices (MinION, Oxford Nanopore) have been deployed to monitor viral outbreaks, including the West African 2013-16 EBOV outbreak [228] and the Lassa fever virus (LASV) outbreak in Nigeria in 2018 [229]. This technology has multiple advantages for pathogen surveillance in real-time when compared to conventional NGS platforms including low cost, portability, easy library preparation, and rapid generation of data in just a few hours [230]. The re-emergence of YFV as a global health burden has been demonstrated by multiple recent outbreaks [15]. In combination with the ION Torrent and Illumina platforms, MinION sequencing was utilized to provide insights into the molecular epidemiology of the 2016–2017 YFV outbreak in Brazil, generating data spatially correlating infections of humans with infection of non-human primates [16]. These data indicate that vector-mediated transmission in the Brazilian outbreak was dominated by continued primate-to-human transmission rather than a human-to-human transfer often observed in explosive African outbreaks. While future outbreaks are inevitable, rapid identification of YFV as the causative agent and subsequent real-time tracking of epidemics can be facilitated by recent advances in NGS technologies and phylogenetics [231], enhancing effective outbreak management, including coordinating vaccination programs and vector eradication campaigns. Additionally, in future outbreaks, viral sampling could be coupled with serological markers of infection (anti-YFV antibodies) and patient DNA bio-banking to enable stratification of host-genetic correlates of YFV infection outcomes, and correlation with host single nucleotide polymorphisms (SNPs). It is likely that host genetics underlie the range of clinical symptoms observed in YFV infection, which include subclinical, symptomatic, severe and fatal outcomes. However, significant challenges to wide-spread implementation of the strategies outlined above should not be underestimated. High costs associated with NGS, coupled with underdeveloped infrastructure in the majority of YFV-endemic countries remain logistical obstacles to overcome.

In addition to diagnosis and outbreak surveillance, the application of NGS to longitudinal and cross-sectional monitoring of infected patient cohorts can facilitate advances in our understanding of within-host viral evolution. Despite near-perfect environmental conditions, including the presence of replication-competent *Aedes* vector species, YFV outbreaks in Asia remain conspicuously absent. However, import of YFV from the Angolan outbreak to China was recorded for the first time in 2016 [15,232]. Using Illumina sequencing to follow virus evolution in 12 imported YFV cases revealed the infecting strains were closely related to each other and possessed limited divergence from the Angola 1971 strain (three amino acid substitutions) despite a 45-year period between samplings [12,233]. The action of strong purifying selection was detected throughout viral genomes, with intra-host variation dominated by synonymous substitutions [233]. In agreement with these observations, the evolutionary rate of YFV has been shown to be lower than that of DENV [234] and the YFV RdRp possesses high fidelity [223]. In addition, sequential bottlenecks associated with vector-host switching could potentially select for variants with an enhanced capacity for transmission, reducing variation. Constraints on YFV evolution could also be mediated by the requirement for interactions with host-dependency factors essential for replication and dissemination in both mammals and arthropods, coupled with a limited duration of infection in human hosts.

### 8.3. Identification of Novel Animal Reservoirs

YFV represents a zoonotic pathogen: while many zoonotic viruses cause severe pathology in humans, tolerance and absence of disease is often observed in reservoir species [235]. After long periods of absence, sporadic and often explosive YFV outbreaks occur in endemic regions, initiated by vector-mediated spillover from jungle primates when environmental conditions are favorable. This pattern of emergence poses the question of whether, in addition to described host- and vector-species, other as yet unidentified animal or arthropod species can act as reservoirs for YFV. Vertical transmission of YFV between mosquitos has been reported [236,237]. While transmission rates are low (~1%), this capacity could maintain YFV reservoirs during dry periods which are not favorable for transmission [4]. It has been suggested that primate density is often not sufficient to maintain stable sylvatic transmission, and YFV reservoirs in non-primate species could maintain inter-epidemic YFV [4]. Indeed, serum neutralizing antibodies for YFV were detected in ten forest mammal species from French Guiana, including members of the divergent orders Rodentia and Carnivora, the superorder Xenarthra, as well as multiple primate species [143]. Thus, the breadth of YFV species-specificity remains to be determined. To further investigate this, extensive sampling and NGS screening of jungle species in YFV endemic regions could be undertaken, potentially identifying novel animal reservoirs which silently maintain YFV transmission between intermittent outbreaks. In summary, future YFV outbreaks can be rapidly identified and epidemics monitored at high resolution using NGS to inform containment strategies. This technology also lends itself to tracking intra-host evolution and the identification of novel animal reservoirs.

## 9. Modes of YFV Transmission and Future Vector Control Strategies

YFV is transmitted by mosquito vectors between primate hosts and transmission to humans in three distinct cycles is possible. 1. The sylvatic or jungle cycle—where epizootic transmission between rainforest primates by jungle-resident mosquito species occurs, with sporadic spillover to forestry workers. This is mediated by multiple *Aedes* species in Africa and *Haemogogus* and *Sabethes* species in South America; 2. The intermediate or savannah cycle—occurs in isolated rural communities on the rainforest periphery where infected mosquito species feed simultaneously on both primate and human hosts. In Africa, *Aedes africanus* preferentially feeds on primates while *Aedes simpsonii* feeds indiscriminately on nonhuman primates and humans; 3. The urban cycle—where invasive domesticated mosquitoes (*Aedes aegypti*) facilitate YFV transmission from human-to-human in areas of high human and vector population density [3,5,6]. The 2015–2016 outbreak in Angola is considered an urban outbreak, with human mobility and population density identified as important factors in the spatial expansion of the epidemic [13]. Contrastingly, detailed analyses of the 2016–2017 Brazilian YFV outbreak suggests the sylvatic cycle represents the dominant mode of transmission [16].

Urban YFV outbreaks are associated with *Ae. aegypti* transmission, although the Asian tiger mosquito, *Aedes albopictus*, is also competent for YFV transmission [238]. Both species are invasive, are able to breed in water containers and have drought-resistant eggs. Indeed, the geographical distribution of these important vector species continues to increase, fueled by human population growth, globalization, increasing urbanization and ongoing climate change [239], expanding the risk of human exposure to YFV infection. The expansion of the 2016–2017 outbreak in Brazil to an area not previously considered endemic for YFV serves to underline this point [15,16]. However, during YFV outbreaks, while isolated cases can be imported from endemic to non-endemic regions [17], continued host-to-host transmission of YFV observed in outbreaks is only possible in geographic regions where competent vector species reside: the disease is not contagious in their absence. This facet of the viral life cycle has been exploited in the past to control and even eradicate YFV from specific regions. While YFV has never been controlled in Africa, vector control programs coupled with the absence of a sylvatic cycle enabled its complete eradication from Europe and North America, where devastating outbreaks were previously common [2,3,4,6]. Vector control programs in South America all but eradicated *Ae. aegypti* by the 1960s [4], although this vector species has re-populated the continent due to the cessation of such programs and remains endemic in 13 countries [240]. In addition, vector resistance to traditional insecticides has been detected in the major invasive *Aedes* vectors and is becoming an increasing problem [7,241,242]. Thus, in combination with traditional chemical spraying of vector breeding sites and vaccination campaigns targeted to at-risk people in endemic regions, innovative new strategies for vector control are required to help to manage and reduce the likelihood of future arbovirus outbreaks, including YFV [243].

Mosquito population suppression strategies which harness the power of genetic modification include the release of insects carrying a dominant lethal gene (RIDL). In trials performed on the Cayman Islands, engineered male *Ae. aegypti* RIDL mosquitos (strain OX513A) carrying a dominant lethal transgene were released, resulting in the suppression of wild mosquito numbers [244]. In a complementary approach using bacteria which are known to render mosquitos sterile, researchers were recently able to virtually eliminate *Ae. albopictus* from two test-release sites in China, combining population suppression and population replacement strategies [245]. Male mosquitoes artificially infected with Wolbachia *w*Pip strains were unable to produce viable offspring after mating with non-*w*Pip infected females, and *w*Pip infected mosquitos of both sexes were less susceptible to infection with the pathogenic flaviviruses DENV and ZIKV [246]. Pupal irradiation boosted viable mosquito numbers for use in the trial ten-fold compared to previous conventional manual inspection techniques, and this combined approach was able to substantially reduce both wild mosquito numbers and biting rates at test-sites [245,246]. While the current limitations of approaches outlined above include associated costs, relatively small trial sizes and isolated trial sites, these exiting results pave the way for analogous trials with *Ae. aegypti* in YFV endemic regions.

Arboviruses often replicate in mosquito mid-gut and targeting this site of viral replication via genetic modification represents another possible strategy for ablating vector-mediated transmission of YFV. Indeed, DENV dissemination and replication were restricted in transgenic *Ae. aegypti* mosquitos ectopically expressing Loqs2, which interacts with intrinsically expressed Loquacious and r2d2 to restore efficient siRNA suppression of viral replication in the mosquito midgut [247]. Furthermore, attenuated YFV-17D is able to replicate in mosquito mid-gut. However, in contrast to wild-type strains, the basal membrane of the midgut blocks YFV-17D dissemination to other tissues [153], indicating a loss of interaction with a co-opted mid-gut host factor during 17D attenuation. In the future, genetic modification of YFV *Aedes* vector species using CRISPR/Cas9 [222] could preferentially target co-opted factors in the mosquito midgut or other tissues which are essential YFV propagation and dissemination. The ability of researchers to identify such factors, which are currently unknown, is enhanced by an improved annotated genome for *Ae. aegypti* [248]. Indeed, these data identified gene loci potentially associated with DENV competence and insecticide resistance. In addition, quantification and comparison of gene expression profiles for distinct *Ae. aegypti* tissues, from both male and female mosquitos at different stages of the reproductive cycle are described [248,249]. Together, these data highlight tissue- and sex-specific gene products, which could be further investigated to identify essential YFV replication cofactors in the mosquito mid-gut via iterative screening approaches. Genetically modified mosquitos with CRISPR/Cas9 targeted deletion of such factors could then be generated for replacement studies. In summary, targeting vector species, which have become resistant to traditional insecticides, will be key in reducing future YFV outbreaks. The future of eliminating YFV from endemic regions may involve the suppression or replacement of wild-type mosquitos with modified insects which are not competent for YFV replication. Furthermore, sequencing efforts to expand the number of annotated genomes from jungle-resident mosquito species should commence.

## 10. Concluding Remarks

YFV is the prototypic flavivirus—a zoonotic pathogen which is responsible for continuing outbreaks of VHF in tropical regions. Recent epidemics in Angola and Brazil serve to underline that the threat from this re-emerging pathogen remains high despite the long-standing availability of a protective vaccine. Scaling back of vector control programs, low vaccine coverage and limitations on vaccine production impedes effective management of outbreaks. Furthermore, rapid global warming, coupled with increasing urbanization and globalization, is expanding permissive mosquito ranges and increasing the potential for future outbreaks. Therefore, intensification of basic YFV research is imperative to expand current knowledge of the complex viral life cycle and stimulate the development of novel anti-YFV therapeutics targeted at both the virus and the host. Additionally, disentangling immune correlates of YFV-induced pathogenesis versus protection, addressing bottlenecks associated with vaccine production, real-time surveillance of outbreaks and genetic modification of vector species to reduce YFV permissiveness can all contribute to future efforts aimed at reducing mortality rates associated with YFV epidemics.

## Supplementary Materials

The following are available online at https://www.mdpi.com/1999-4915/11/10/960/s1, Table S1: Summary of structural and functional properties of YFV proteins.
