# Supplementary material for: Yellow Fever: Integrating Current Knowledge with Technological Innovations to Identify Strategies for Controlling a Re-Emerging Virus
Source: Viruses. 2019 Oct 17;11(10):960. doi: 10.3390/v11100960 (PMC6832525; doi:10.3390/v11100960)
Supplement: Supplementary file 1 [file viruses-11-00960-s001.pdf]

Supplementary table 1. Summary of structural and functional properties of YFV proteins.

Flavivirus Proteins	Type of Protein and/or Sequence Elements	Functions	Interaction with Host/Viral proteins or nucleotide sequences	Functional relation to the Immune system	References
Capsid	14-kDa Capsid protein	• Packing the viral RNA<br>• Nucleocapsid core (1)			(1) [1]
prM	25-kDa type I membrane protein	• To inhibit the preactivation of E protein during the transport from ER to the trans-Golgi network (1)			(1) [2]
M	8-kDa type I membrane protein	• To protect the viral particle<br>• To form 1:1 complex with E protein (1)			(1) [3]
E protein	52-kDa type I membrane protein	• Attachment to target cells<br>• Interaction with surface receptors<br>• Fusion of the viral particle with endosomal membrane (1)		• Immunogenic region to induce neutralizing antibodieds response (2)	(1) [4]<br>(2) [5]
NS1	48-kDa Multifunctional glycoprotein (monomer, dimer (membrane-bound protein, mNS1), and a hexamer (secreted protein, sNS1))	• Localized in the intracellular membranes or at the cell surface (1)<br>• Viral replication (2)<br>• Cofactor for NS5 (3)<br>• Remodelling the ER membrane for vesicle packets (VPs) (3)<br>• To trigger the endothelial permeability (4)	• C4 and C1s (Complement) (5)<br>• STAT3 beta (8)	• To activate the TLR and inhibit the complement system (immune invasion) (6, 7)	(1) [6]<br>(2) [7]<br>(3) [8]<br>(4) [9]<br>(5) [10]<br>(6) [11]<br>(7) [12]<br>(8) [13]

NS2A	22-25-kDa Integral hydrophobic membrane protein	• Replication complex (1)<br>• Assembly (1, 2)<br>• The N-terminus is processed by membrane-bound host protease in the ER and C-terminus by NS2B-NS3 viral protease (3)<br>• To enhance membrane permeability (5)	• Interaction with the 3'UTR of the viral RNA (4)<br>• NS4A, NS3, and NS5 (4)<br>•		(1) [14]<br>(2) [15]<br>(3) [16]<br>(4) [17]<br>(5) [18]
NS2B	14-kDa integral membrane protein	• Cofactor for protease activity of NS3 (1)	• NS3 (1)		(1) [19]
NS3	69-kDa multifunctional protein<br>• RNA triphosphatase (RTPase)<br>• Nucleoside 5' triphosphatase (NTPase)<br>• RNA helicase<br>• Serine protease	• Replication complex (1)<br>• Protease activity (with NS2B) (1)	• NS2B (1)		(1) [20]
NS4A	16-kDa integral membrane protein	• Remodeling the ER membrane for vesicle packets (VPs) (1)<br>• Replication complex (2)	• Viral dsRNA (3)<br>• NS1, NS2A, and NS5 (3)	• Autophagy (4)	(1) [21]<br>(2) [22]<br>(3) [23]<br>(4) [24]
NS4B	30-kDa integral membrane protein	• Cleaved by peptide 2K (1)<br>• Remodelling the ER membrane for vesicle packets (VPs) (2)<br>• Helicase activity of NS3 (3)	• NS3, NS1, and NS4A (1)<br>• STING (5)	• Suppression the α/β interferon signaling (4)<br>• To inhibit RIG-1 by STING binding (5)	(1) [25]<br>(2) [20]<br>(3) [26]<br>(4) [27]<br>(5) [28]

NS5	105-kDa multifunctional protein<br>• Methyltransferesase<br>• Guanylyltransferase<br>• RNA-dependent RNA polymerases (RdRp)	• Replication complex (1)<br>• Capping the nascent viral RNA (1)	• NS2A, NS3, and NS4A (1)<br>• STAT2 (2)	• Inhibitor of IFN-I signal pathway (2)	(1) [20]<br>(2) [29]

References

1. Patkar, C.G.; Jones, C.T.; Chang, Y.-h.; Warrier, R.; Kuhn, R.J. Functional requirements of the yellow fever virus capsid protein. *J. Virol.* **2007**, *81*, 6471–6481, doi:10.1128/JVI.02120-06.
2. Guirakhoo, F.; Heinz, F.X.; Mandl, C.W.; Holzmann, H.; Kunz, C. Fusion activity of flaviviruses: comparison of mature and immature (prM-containing) tick-borne encephalitis virions. *J. Gen. Virol.* **1991**, *72 (Pt 6)*, 1323–1329, doi:10.1099/0022-1317-72-6-1323.
3. Li, L.; Lok, S.-M.; Yu, I.-M.; Zhang, Y.; Kuhn, R.J.; Chen, J.; Rossmann, M.G. The flavivirus precursor membrane-envelope protein complex: structure and maturation. *Science* **2008**, *319*, 1830–1834, doi:10.1126/science.1153263.
4. Zhang, Y.; Zhang, W.; Ogata, S.; Clements, D.; Strauss, J.H.; Baker, T.S.; Kuhn, R.J.; Rossmann, M.G. Conformational changes of the flavivirus E glycoprotein. *Structure* **2004**, *12*, 1607–1618, doi:10.1016/j.str.2004.06.019.
5. Kielian, M. Class II virus membrane fusion proteins. *Virology* **2006**, *344*, 38–47, doi:10.1016/j.virol.2005.09.036.
6. Muller, D.A.; Young, P.R. The flavivirus NS1 protein: molecular and structural biology, immunology, role in pathogenesis and application as a diagnostic biomarker. *Antiviral Res.* **2013**, *98*, 192–208, doi:10.1016/j.antiviral.2013.03.008.
7. Mackenzie, J.M.; Jones, M.K.; Young, P.R. Immunolocalization of the dengue virus nonstructural glycoprotein NS1 suggests a role in viral RNA replication. *Virology* **1996**, *220*, 232–240, doi:10.1006/viro.1996.0307.
8. Apte-Sengupta, S.; Sirohi, D.; Kuhn, R.J. Coupling of replication and assembly in flaviviruses. *Curr. Opin. Virol.* **2014**, *9*, 134–142, doi:10.1016/j.coviro.2014.09.020.
9. Beatty, P.R.; Puerta-Guardo, H.; Killingbeck, S.S.; Glasner, D.R.; Hopkins, K.; Harris, E. Dengue virus NS1 triggers endothelial permeability and vascular leak that is prevented by NS1 vaccination. *Sci. Transl. Med.* **2015**, *7*, 304ra141, doi:10.1126/scitranslmed.aaa3787.
10. Avirutnan, P.; Fuchs, A.; Hauhart, R.E.; Somnuke, P.; Youn, S.; Diamond, M.S.; Atkinson, J.P. Antagonism of the complement component C4 by flavivirus nonstructural protein NS1. *J. Exp. Med.* **2010**, *207*, 793–806, doi:10.1084/jem.20092545.
11. Young, P.R.; Hilditch, P.A.; Bletchly, C.; Halloran, W. An antigen capture enzyme-linked immunosorbent assay reveals high levels of the dengue virus protein NS1 in the sera of infected patients. *J. Clin. Microbiol.* **2000**, *38*, 1053–1057.
12. Macdonald, J.; Tonry, J.; Hall, R.A.; Williams, B.; Palacios, G.; Ashok, M.S.; Jabado, O.; Clark, D.; Tesh, R.B.; Briese, T.; et al. NS1 protein secretion during the acute phase of West Nile virus infection. *J. Virol.* **2005**, *79*, 13924–13933, doi:10.1128/JVI.79.22.13924-13933.2005.
13. Chua, J.J.-E.; Bhuvanakantham, R.; Chow, V.T.-K.; Ng, M.-L. Recombinant non-structural 1 (NS1) protein of dengue-2 virus interacts with human STAT3beta protein. *Virus Res.* **2005**, *112*, 85–94, doi:10.1016/j.virusres.2005.03.025.
14. Kümmerer, B.M.; Rice, C.M. Mutations in the yellow fever virus nonstructural protein NS2A selectively block production of infectious particles. *J. Virol.* **2002**, *76*, 4773–4784, doi:10.1128/jvi.76.10.4773-4784.2002.

15. Leung, J.Y.; Pijlman, G.P.; Kondratieva, N.; Hyde, J.; Mackenzie, J.M.; Khromykh, A.A. Role of nonstructural protein NS2A in flavivirus assembly. *J. Virol.* **2008**, *82*, 4731–4741, doi:10.1128/JVI.00002-08.
16. Falgout, B.; Pethel, M.; Zhang, Y.M.; Lai, C.J. Both nonstructural proteins NS2B and NS3 are required for the proteolytic processing of dengue virus nonstructural proteins. *J. Virol.* **1991**, *65*, 2467–2475.
17. Mackenzie, J.M.; Khromykh, A.A.; Jones, M.K.; Westaway, E.G. Subcellular localization and some biochemical properties of the flavivirus Kunjin nonstructural proteins NS2A and NS4A. *Virology* **1998**, *245*, 203–215, doi:10.1006/viro.1998.9156.
18. Chang, Y.S.; Liao, C.L.; Tsao, C.H.; Chen, M.C.; Liu, C.I.; Chen, L.K.; Lin, Y.L. Membrane permeabilization by small hydrophobic nonstructural proteins of Japanese encephalitis virus. *J. Virol.* **1999**, *73*, 6257–6264.
19. Choksupmanee, O.; Hodge, K.; Katzenmeier, G.; Chimnaronk, S. Structural platform for the autolytic activity of an intact NS2B-NS3 protease complex from dengue virus. *Biochemistry* **2012**, *51*, 2840–2851, doi:10.1021/bi2018267.
20. Lindenbach, B.D. Virion Assembly and Release. *Curr. Top. Microbiol. Immunol.* **2013**, *369*, 199–218, doi:10.1007/978-3-642-27340-7_8.
21. Miller, S.; Kastner, S.; Krijnse-Locker, J.; Bühler, S.; Bartenschlager, R. The non-structural protein 4A of dengue virus is an integral membrane protein inducing membrane alterations in a 2K-regulated manner. *J. Biol. Chem.* **2007**, *282*, 8873–8882, doi:10.1074/jbc.M609919200.
22. Roosendaal, J.; Westaway, E.G.; Khromykh, A.; Mackenzie, J.M. Regulated cleavages at the West Nile virus NS4A-2K-NS4B junctions play a major role in rearranging cytoplasmic membranes and Golgi trafficking of the NS4A protein. *J. Virol.* **2006**, *80*, 4623–4632, doi:10.1128/JVI.80.9.4623-4632.2006.
23. Khromykh, A.A.; Westaway, E.G. Completion of Kunjin virus RNA sequence and recovery of an infectious RNA transcribed from stably cloned full-length cDNA. *J. Virol.* **1994**, *68*, 4580–4588.
24. McLean, J.E.; Wudzinska, A.; Datan, E.; Quaglino, D.; Zakeri, Z. Flavivirus NS4A-induced autophagy protects cells against death and enhances virus replication. *J. Biol. Chem.* **2011**, *286*, 22147–22159, doi:10.1074/jbc.M110.192500.
25. Welsch, S.; Miller, S.; Romero-Brey, I.; Merz, A.; Bleck, C.K.E.; Walther, P.; Fuller, S.D.; Antony, C.; Krijnse-Locker, J.; Bartenschlager, R. Composition and three-dimensional architecture of the dengue virus replication and assembly sites. *Cell Host Microbe* **2009**, *5*, 365–375, doi:10.1016/j.chom.2009.03.007.
26. Umareddy, I.; Chao, A.; Sampath, A.; Gu, F.; Vasudevan, S.G. Dengue virus NS4B interacts with NS3 and dissociates it from single-stranded RNA. *J. Gen. Virol.* **2006**, *87*, 2605–2614, doi:10.1099/vir.0.81844-0.
27. Muñoz-Jordán, J.L.; Laurent-Rolle, M.; Ashour, J.; Martínez-Sobrido, L.; Ashok, M.; Lipkin, W.I.; García-Sastre, A. Inhibition of alpha/beta interferon signaling by the NS4B protein of flaviviruses. *J. Virol.* **2005**, *79*, 8004–8013, doi:10.1128/JVI.79.13.8004-8013.2005.
28. Ishikawa, H.; Ma, Z.; Barber, G.N. STING regulates intracellular DNA-mediated, type I interferon-dependent innate immunity. *Nature* **2009**, *461*, 788–792, doi:10.1038/nature08476.
29. Laurent-Rolle, M.; Morrison, J.; Rajsbaum, R.; Macleod, J.M.L.; Pisanelli, G.; Pham, A.; Ayllon, J.; Miorin, L.; Martinez, C.; tenOever, B.R.; et al. The interferon signaling antagonist function of yellow fever virus NS5 protein is activated by type I interferon. *Cell Host Microbe* **2014**, *16*, 314–327, doi:10.1016/j.chom.2014.07.015.
